# Prevention and Treatment of COVID-19 Using Traditional and Folk Medicine: A Content Analysis Study

**DOI:** 10.4314/ejhs.v31i6.3

**Published:** 2021-11

**Authors:** Nazi Nejat, Ali Jadidi, Ali Khanmohamadi Hezave, Seyed Mohamad Aghae Pour

**Affiliations:** 1 PhD in Nursing, Assistant Professor. School of Nursing, Arak University of Medical Sciences, Arak, Iran; 2 PhD in Nursing, Assistant Professor. Traditional and Complementary Medicine Research Center, Arak University of Medical Sciences, Arak, Iran; 3 PhD in Nursing, School of Nursing, Arak University of Medical Sciences, Arak, Iran; 4 BS in Nursing, Arak University of Medical Sciences, Arak, Iran

**Keywords:** COVID-19, Folk Remedies, Traditional medicine, Prevention

## Abstract

**Background:**

Coronavirus 2019 (COVID-19) is a respiratory disease with no specific and definitive drug treatment. With the COVID-19 outbreak, traditional and folk methods were used for its treatment. This study was conducted to explore people's experiences of using traditional and folk medicine for the prevention and treatment of COVID-19 in Iran.

**Methods:**

This qualitative study was conducted on 37 people in Arak (Iran) from May to November 2020 using a conventional content analysis approach. The participants were selected using cluster sampling and interviewed using semistructured telephone interviews. After transcribing the interviews, they were analyzed using content analysis. Accordingly, semantic units were identified, related codes were extracted and placed in subcategories and categories based on similarities, and themes were formed. The interviews continued until data saturation.

**Results:**

After analyzing the collected data, 116 different codes were extracted and classified into two subcategories of pharmacological and non-pharmacological methods. Then, each of the subcategories was classified into two categories: conventional medicine and traditional medicine. Finally, two main themes were obtained, including prevention methods and self-treatment methods for COVID-19.

**Conclusion:**

People use various traditional and folk methods for COVID-19 prevention and treatment. Such methods can be either useful or lack the necessary effectiveness and have side effects. Thus, necessary training should be provided to the public about using these methods and avoiding unapproved treatments.

## Introduction

Coronavirus 2019 (COVID-19) is a new infectious disease that results in acute respiratory syndrome caused by Coronavirus 2. The first COVID-19 case was reported in December 2019, and the disease became a global pandemic afterward (1). COVID-19 has also affected Iran, incurring huge socioeconomic costs.

Like many other respiratory viruses, COVID-19 is transmitted through contacting eyes, nose, and mouth with hands infected with respiratory secretions, respiratory droplets due to sneezing, coughing, and even long face-to-face dialogues (2). The most significant disease symptoms are headache, sore throat, runny nose, fever and persistent cough. In more severe cases, the disease can cause severe respiratory symptoms and death. Epidemiological and clinical examinations have been performed to find an effective treatment for the disease (3). The World Health Organization's specialized committees have supported the development of traditional medicine-based treatments against COVID-19 and the implementation of approved protocols to produce scientific evidence on the quality, safety, and effectiveness of herbal medicines for the disease (4).

However, there are no definitive and effective treatments for the disease (3). Here, some traditional medicines and folk medicine are used arbitrarily or prescribed by physicians to prevent and treat the disease. Folk medicine and traditional medicines are a sub-branch of complementary and alternative medicine (5), including psychosocial interventions, herbs, ritual behaviors, or folk medicines recommended by various individuals or groups in society or religious groups (6). Although most folk medical treatments are relatively harmless, their use is still controversial in the scientific literature. It should be noted that over 80% of the population in developing countries use traditional therapies for their primary healthcare (7).

Given its long history and different ethnicities, races, languages, and climates, Iran is full of folk medical experiences and beliefs. However, studies indicate that the available information on folk medicine of various ethnicities and cultures is not well documented. Delays in documenting this information leads to the loss of some of these valuable experiences; the ones that may be the key to solving some medical problems (8). In a study on beliefs in using home and folk remedies in treating diseases, 59% of women under study had a high level of knowledge and firm beliefs in this regard (9). In another study, a significant percentage of women in all socioeconomic classes were using home remedies (10).

However, using these methods without a healthcare team's supervision and approval could interfere with medical treatments or cause various complications. For instance, in March 2019, the arbitrary decision to drink alcohol to protect against COVID-19 poisoned and killed several people in Iran (11). Furthermore, there are no official statistics on the type and extent of use of these treatments in Iran. Clearly, evaluating the attitude and status of using various folk medicine and traditional medicines in various regions, one can extract health policies and general and regional strategies for the proper use of these treatments and minimize their complications (12). Healthcare systems could have an influential role in achieving therapeutic goals by identifying various home and folk medicine used by families, providing necessary training, and preventing complications caused by the unscientific use of these treatments. Thus, this study was conducted to qualitatively explanation folk medicine and traditional approaches used for COVID-19 prevention and treatment in Arak (Iran).

## Materials and Methods

**Study design**: This qualitative study was conducted using conventional content analysis from May to November 2020 in Iran.

**Participants**: The participants were 37 citizens selected using purposive and cluster sampling methods from May to November 2020 (according to the four geographical areas of Arak and by using telephone numbers received from a health center). Sampling was continued until data saturation, meaning that no new codes were extracted from the transcripts. In the sampling process, the maximum diversity regarding age, gender, education, and occupation was considered to increase the data richness. The inclusion criteria were having a positive test of COVID-19 among individuals or their family members, living in Arak, being over 18 years of age, and speaking Persian. Table 1 shows the participants' demographic characteristics.

**Table 1:** Demographic characteristics of the participants

Age	Mean and standard deviation: 46.4 ± 7.81 years
**Gender**	male (16 people), female (21 people)
**Marital** **status**	Married (29 people), Single (6 people), Widower (2 people)
**Degree of** **education**	illiterate (1 person), elementary (9 people), high school diploma (16 people), bachelor's (7 people), master's (3 people), Ph.D. (1 person)
**Employment**	housewife (14 people), employee (9 people), teacher (2 people), retired (6 people), self-employed (6 people)
**Having** **insurance**	yes (33 people), no (4 people)
**Having** **COVID-19**	yes (8 people), no (29 people)

**Data collection**: The primary data collection method was semi-structured interviews conducted by telephone when the participants were comfortable. The interviews began by asking some warm-up questions to gain the participants' trust. Then, general questions were asked, like “what traditional and folk medicine methods have you used to prevent or treat COVID-19?” We continued the interview with exploratory questions like “can you explain more” or “when you say..., what do you mean?”, if necessary. At the end of the interview, the participants were asked; if there was anything else, they wanted to share. The interviews were conducted in one or more sessions depending on the participants' time and patience, their willingness, and the obtained information. Each interview session lasted between 30 and 45 min, depending on the participant's conditions and patience, with the average of 39.4±3.5 min. The interviews were recorded with the participants' consent and then transcribed verbatim.

**Data analysis**: In this study, data analysis was performed using conventional content analysis with MAXQDA10 software. In the content analysis method, the researcher analyzed the generated messages and attempted to find answers to the research questions (13). Thus, after the careful study of the transcripts, semantic units were identified. Then, relevant codes were extracted, and based on their similarities; they were classified into subcategories and categories. Finally, themes were obtained.

**Rigour**: Guba and Lincoln proposed four criteria for the accuracy and robustness of qualitative data (14). In this study, the data was validated by peers and reviewed by the participants through long-term engagement with the data and with sufficient time to collect and analyze the data. The data transferability was evaluated by verifying the information obtained by two non-researchers with similar positions to the study participants. For validation, the researcher carefully recorded and reported the research process and the decision path, allowing others to follow up on the research. Subsequently, two nursing experts at the School of Nursing used the external observer method to examine the similarity of their understanding with the researcher and search for conflicting cases.

**Ethical considerations**: The study is part of a project approved by the Ethics Committee of the Arak University of Medical Sciences (code IR.ARAKMU.REC.1399.013). Before the interview, the participants were informed of the study's purpose and the interview method, and oral consent was obtained. The participants' privacy and confidentiality were protected in all stages of the research. They also had the right to leave the study at any time.

## Results

After analyzing the collected data, 116 various codes were extracted and classified into two subcategories of pharmacological and non-pharmacological methods. Then, each of the subcategories was classified into two categories: conventional medicine and traditional medicine. Ultimately, two main themes were formed, including prevention methods and treatment methods for COVID-19, as explained below.

## A. Prevention Methods

**1. Conventional medicine**: This category, with two subcategories of pharmacological and nonpharmacological methods, had all the measures that the participants took to prevent COVID-19 using conventional methods. In the pharmacological methods subcategory, the participants used influenza vaccines, various vitamins, and cold pills. In this regard, one of the participants stated:

*“I received the flu vaccine not to catch the flu for a while. I might get corona because my body may be weak”* (A 27-year-old woman).

Another participant stated the significance of taking supplements, especially vitamins, in the prevention of this disease:

*“I heard that vitamin D is very good for prevention. I try to take one vitamin D pill a month. I also got a vitamin C pill to take daily”* (A 45-year-old woman).

In the non-pharmacological subcategory, the participants mentioned many of the items frequently reported by national or social media. Maintaining social distance, washing and disinfecting hands, washing and disinfecting objects and food, using safety measures like masks and gloves, checking body temperature regularly, and chewing gum were the most significant measures in this subcategory.

Regarding this, one of the participants pointed out:

*“I have never gone out without a mask or gloves since the onset of the illness and I only go out for emergencies. I try to get out of the house as softly as possible”* (A 67-year-old man).

While emphasizing maintaining social distance, another participant stated:

*“I did not go to anyone's house for several months and no guests came to our home. I called the house of all our relatives and told them that we should only communicate with each other by phone or virtually during the disease”* (A 42-year-old woman).

While emphasizing food hygiene, another participant mentioned:

*“I always disinfect fruits and vegetables with a few drops of dishwashing liquid in a basin of water for 4 to 5 minutes to kill the corona virus”* (A 46-year-old woman).

**2. Traditional and folk medicine:** This category had two subcategories of pharmacological and non-pharmacological methods and included all the measures participants used to prevent COVID-19 using traditional and folk medicine methods.

In the pharmacological subcategory, using various herbal medicines like incense, infusion, and chewing or sucking was emphasized. Thyme incense was one of the most significant items mentioned by a significant number of the participants, and it was considered useful in preventing this disease. Moreover, using different teas like thyme, borage, fennel, mountain tea, orange blossom, and chamomile was suggested by almost all the participants. One of the participants claimed:

*“I always use thyme, mountain tea and chamomile, but I have been taking more than ever since this illness [Corona] became common. I think it is very effective”* (A 52-year-old man).

Describing the extraordinary effect of honey syrup on preventing this disease, one of the participants stated:

*“I have the experience of honey syrup, of course, if it is natural honey with natural lemon juice is very good for diseases like colds and COVID-19, especially to prevent this disease, it should always be used”* (A 39-year-old man).

Moreover, using garlic and onion with food, consuming black seed, cinnamon, jujube, chewing frankincense, and sucking licorice candy were other items used by the participants to prevent the disease. One of the participants expressed:

*“I heard that the throat should not be dry to prevent this disease. I always put frankincense or licorice chocolate in my mouth so that both the throat does not dry out and that these drugs themselves have an antiseptic role”* (A 32-year-old woman).

In the non-pharmacological subcategory, the participants mentioned measures including disinfecting hands and surfaces with herbal compounds like thyme decoction or thyme spray and using healing practices like taking a hot bath, cupping, and using bee sting. Emphasizing the role of hand and surface disinfection, one of the participants stated:

*“Hands and objects have to be disinfected regularly. However, I do not use chemicals as it may lead to allergies. I use thyme spray for hand and surface disinfection. If it is not, I boil thyme and smooth it with my hands and disinfect other things”* (A 28-yearold woman).

Another participant stressed the effect of cupping on preventing many diseases, including COVID-19, and pointed out:

*“Cupping really makes wonders. It has a great effect on preventing diseases. I have been catching a cold for a few years now. I have very few colds. It is very mild. I am sure that it also has a great effect on preventing corona”* (A 48-year-old man).

Another participant highlighted the significance of bee stings:

*“I have heard from the elderly that bee stings have many properties also effective in preventing corona, so I go to places where there are bees to bite”* (A 34-year-old man).

Another participant said:

*“I do everything they can to prevent coronation, but I also prayed and vowed that my family and myself would not get the disease, said one participant, emphasizing the role of vows in preventing coronation. I believe that everything is in God's hands.”* (A 50-year-old woman)

## B. Self-Healing Methods

**1. Conventional medicine:** This subcategory included all the pharmacological and nonpharmacological items the participants used to treat or alleviate COVID-19 symptoms in case they or those around them were infected. The participants also reported using items including hydroxychloroquine tablets, oxygen, expectorant syrup, analgesics, and antipyretics like acetaminophen, codeine, ibuprofen, and aspirin. Some of these items were prescribed by a doctor, and people used similar prescriptions for the other items. In many cases, the patients made arbitrary decision to use the items. One of the participants recently recovering from the disease stated:

*“When my test was positive, the doctor told me that there was no need to be hospitalization and rest at home and that there is no need for any special medicine. He just said to have oxygen at home and use if you suffer from shortness of breath. There was no need to use oxygen, but because I had pain and fever, I used acetaminophen codeine and ibuprofen every few hours to reduce pain and fever”* (A 35-year-old man).

The participants also frequently used non-pharmacological treatment methods, often recommended by the media or treatment staff and physicians. Quarantining patients, using masks and gloves, frequently washing clothes and sheets, gargling salt water, consuming hot liquids, resting, and eating hot soup were among the measures stated in the interviews. One of the participants mentioned:

*“I followed the entire doctor's advice. I locked myself in a room for up to two weeks. I had no contact with anyone. I washed my hands regularly, gargled salt water, wore a mask and gloves, drank hot liquids until I finally recovered”* (A 29-year-old male).

However, the participants also used some other methods. One of them stated:

*“I already knew about relaxation. When my test was positive, I used this method along with the medications I was taking. It was beneficial in reducing stress and anxiety, as well as reducing my headaches and body aches”* (A 28-year-old woman).

Regarding the use of non-pharmacological treatment methods, another participant pointed out:

*“I think the disease, like other viral diseases, needs rest and eating and eating nutritious foods and hot liquids. I did all this for two weeks and got well”* (A 56-year-old man).

**2. Traditional and folk medicine:** In this subcategory, there were pharmacological and non-pharmacological treatments prescribed by non-physicians skilled in traditional medicine, recommended by people slightly familiar with the medicine, or advertised in the media or cyberspace. In pharmacological treatment, items like honey and fresh lemon syrup, thyme powder, cinnamon powder, rose, fresh lemon juice, quadruple glaze, and traditional teas like thyme tea, mountain tea, cinnamon, ginger, hyssop, thistle, turmeric, and chamomile were mentioned by the participants. One of the participants stated:

*“I consulted an herbalist and he told me that hyssopus and echinacea angustifolia tea are very good for treating corona. I took it and used it for a few days. Reduced respiratory symptoms like shortness of breath and cough reduced so much. I think it helped a lot to make our illness better soon”* (A 39-year-old man).

In traditional non-pharmacological treatment, the participants introduced measures like using hot foods, limiting the consumption of cold foods, tying a handkerchief around the head and neck, using a hot hairdryer, warming the body, burning incense, using a garlic necklace, and using smoking methods like inhaling donkey droppings, smoking cigarettes, and smoking cotton (or cotton cloth) indoors. One of the participants stated in this regard:

*“I experienced this myself. I told anyone just smoke a little cotton cloth or a little cotton in the house to eradicate this disease. If you do this task every day, there will be no sign of the disease. His father had corona. I told someone whose father had corona to do that, and a few days later he came and thanked him, saying that it made him feel ok”* (A 69-year-old man).

About non-pharmacological methods, another participant said:

*“When my wife got sick, I was very worried, especially because I knew that this disease was incurable and dangerous. I did everything the doctor said, but I also prayed, and my wife and children and I relied just on God. Thank God everything went well and we all recovered”* (A 51-year-old woman).

Concerning the side effects of using preventive and therapeutic methods, the participants reported no other side effects except for three minor skin and eye disorders while using hydroxychloroquine tablets. A summary of the results is presented in Table 2.

**Table 2:** Summary of the themes and categories extracted from the transcripts

Theme	Category	Subcategory	Codes
Prevention methods	Conventional medicine	Pharmacological methods	Injecting influenza vaccines, taking cold pills, taking vitamins
		Non- pharmacological methods	Maintaining social distance, washing and disinfecting hands, washing and disinfecting objects and food, wearing masks and gloves, opening doors and pressing elevator buttons with paper towels, covering mouth and nose while sneezing and coughing, heating bread before eating, not purchasing unpackaged food, checking body temperature, eating more fresh fruits and vegetables, drinking more water and fluids, chewing gum, quarantining
	Traditional and folk medicine	Pharmacological methods	Smoking thyme, using honey and sour lemon syrup, eating garlic, eating ginger with yogurt, chewing frankincense, using black seed, eating licorice candy, jujube, eating onion and raw garlic with food, consuming various types of tea (borage, thyme)
		Nonpharmacological methods	disinfecting hands with herbal compounds, healing practices like cupping and bee stings, praying
Self- treatment methods	Conventional medicine	Pharmacological methods	taking hydroxychloroquine tablets, using oxygen, consuming expectorant syrup, using antipyretic and analgesic
		Non- pharmacological methods	doing chest physiotherapy, regular washing of hands, quarantining, wearing masks, frequent washing of clothes and sheets, saltwater spools, consuming warm fluids, resting, consuming nutritious foods, relaxing
	Traditional and local medicine	Pharmacological methods	consuming fresh sour honey and lemon syrup, using thyme powder with food, using cinnamon powder with food, using rose, drinking fresh lemon juice, quadruple glaze, consuming the honey and cinnamon combination, incense of baking soda, and using types of tea
		Non- pharmacological methods	smoking (smoking peganum harmala, cigarettes, and cotton, as well as inhaling donkey droppings), using hot foods, limiting the consumption of cold foods, tying handkerchiefs around the head and neck, using a hot hairdryer, warming the body, burning incense, and using a garlic necklace

## Discussion

The results were categorized into two main themes of prevention methods and self-treatment methods, and each had two categories of standard medicine and traditional medicine. Moreover, two subcategories of pharmacological and non-pharmacological methods were revealed in each category.

In a study comparing the effectiveness of Chinese and Western medicine on the treatment of COVID-19 symptoms, Qiu et al. (2020) indicated that both treatments effectively relieved the mild and standard level symptoms. However, the both treatments were ineffective in moderate and severe levels of the disease. Hence, the researchers suggested that complementary and alternative treatments should be carried out according to the disease's severity and the standard protocols; otherwise, it can have adverse results (14).

The participants stated using different medicinal plants in various ways like drinking, burning, chewing, and accompanying to prevent or treat COVID-19. A meta-analysis confirmed the effectiveness of many Chinese treatments like acupuncture, acupressure, yoga, aromatherapy with Chinese perfumes, bamboo incense, ginseng infusion, and various combination drugs (15). In a systematic review, Fan et al. (2020) examined seven clinical trials on 736 patients with COVID-19. Based on the evidence, they concluded that Chinese herbal medicine helped improve treatment outcomes in COVID-19 cases as adjunctive therapy with standard care. Moreover, they reported positive results of using Chinese herbal remedies to enhance patients' clinical and paraclinical symptoms, like a reduction in inflammatory factors and a significant change in the appearance of lung CT scans (16). In a clinical trial, Lin et al. (2020) performed traditional Chinese medicine rehabilitation (TCMR) program, acupressure, and exercise Liuzijue Qigong on 120 patients with COVID-19. The results indicated that more than 60% of the patients completely recovered. Therefore, these researchers suggest that these therapies can improve patients with severe forms of the disease (17). Another study in China (2020) showed that TCMR effectively prevented COVID-19 and that iatrogenic infection could be prevented by consuming a decoction based on TCMR principles (18).

Another method used in the prevention and treatment of this disease, as expressed by the participants, was smoking methods. Smoking cotton and cigarettes and inhaling donkey dropping were some of the issues raised in this regard. Zhou et al. (2020) indicated that smoking drugs and acupressure was the most common use of traditional Chinese medicine in COVID-19 nursing interventions for discharged patients (19).

Moreover, other standard folk medicines used by the participants in this study were cupping therapy, acupressure, and acupuncture. Zhou et al. stated that Gua Sha, limb reflexology, acupuncture, and fumigation were discussed by participants (19). Lin et al. (2020) presented some standard recommendations based on Chinese medicine in a study entitled ‘Traditional Chinese Nursing Protocols for COVID-19’ to relieve various disease symptoms. Ear poultices, acupuncture, special exercises in Chinese medicine, and massage therapy were some of these suggestions (17).

In the present study, the participants stated that using self-medication and preventive methods effectively accelerated the recovery of COVID-19 or prevented the disease. The results of a review paper on traditional medicines recommended for the treatment of COVID-19 showed that traditional Chinese, Indian, and Iranian medicines were considered and some plants were recommended for the prevention, treatment, and rehabilitation of diseases, such as COVID-19 (20). On the contrary, a study by Nugraha et al. (2020) indicated that although many people believed in the effectiveness of traditional medicinal compounds in viral diseases, including COVID-19, laboratory studies rejected many of these beliefs; studies are still being conducted on this area (1). Moreover, in some countries such as Vietnam, Cambodia, Indonesia, Malaysia, Thailand, and the Philippines, different treatments, including boiled lemongrass, boiled garlic juice, garlic juice, and coconut oil, are circulating, especially on the Internet and social media, although they have not been proven to be true (21). Since there are contradictory data on some medicinal plants, it is suggested not to use supplements containing herbal compounds to prevent COVID-19 without a doctor's direct supervision. Additionally, physicians should prescribe herbal medicines with more caution for treating patients with COVID-19 (22, 23) and refrain from prescribing herbal medicines to patients if their effectiveness has not been proven.

It is not common to use traditional and folk treatments in conventional clinics in some countries, and people use these preventive treatments based on unreliable resources. Healthcare providers should know these methods to guide people properly and introduce credible sources to them. Furthermore, many traditional and folk methods have side effects and cause drug interactions. Healthcare providers must have the correct information about complementary and alternative therapies to diagnose their interactions and side effects so that they can provide low-risk methods to enhance client health (24). Healthcare providers also need to evaluate patients for different interactions and side effects and consider whether complementary and alternative therapies could help them by asking questions like, ‘how does the patient understand the treatment? or is this treatment safe? (25).

The findings indicated that the participants also used religious practices to prevent and treat COVID-19. The results of a review study indicated that spirituality could help people have peace of mind in critical situations and in case of dangerous diseases. Some of the religious solutions offered against the prevalence of COVID-19 can be helpful. Hence, medical staff in hospitals have to consider patients' spiritual and religious beliefs with COVID-19 to enhance their comfort and well-being (26,27). In Tanzania, churches and mosques encouraged people to follow instructions issued by the Ministry of Health. Nonetheless, they prayed to God to save their society and country from the virus (22). More clinical studies should be conducted to reveal the favorable effects and disadvantages of traditional and folk medicines (28, 29).

According to the results, people used various traditional and folk medicine methods to prevent and treat OVID-19. Some of the suggested methods can be useful, while others may not be effective or may have significant side effects. Hence, it is suggested to provide people and health workers with training to use reliable methods and avoid unapproved methods.
